# Genetic Diversity and Differentiation Among Guatemalan Cardamom (*Elettaria cardamomum* (L.) Maton) Accessions

**DOI:** 10.3390/plants15040655

**Published:** 2026-02-20

**Authors:** Martha Patricia Herrera-González, Lizbeth Coxaj, Ana Oliva, Margarita Palmieri, Alejandra Zamora-Jerez, Rolando Cifuentes-Velasquez, Santiago Pereira-Lorenzo

**Affiliations:** 1 Center for Agricultural and Food Studies, Universidad del Valle de Guatemala, Guatemala City 01015, Guatemala; lkcoxaj@uvg.edu.gt (L.C.); agoc1994@gmail.com (A.O.); palmieri@uvg.edu.gt (M.P.); rcifuen@uvg.edu.gt (R.C.-V.); 2 Programa de Doctorado en Agricultura y Medio Ambiente para el Desarrollo, Universidade de Santiago de Compostela, 27002 Lugo, Spain; 3 Center for Biotechnology Studies, Universidad del Valle de Guatemala, Guatemala City 01015, Guatemala; oazamora@uvg.edu.gt; 4 Department of Crop Production and Engineering Projects, Escola Politécnica Superior de Enxeñaría, Campus Terra, Universidade de Santiago de Compostela, 27002 Lugo, Spain; santiago.pereira.lorenzo@usc.es

**Keywords:** genetic profiling, genetic differentiation, *Elettaria cardamomum* (L.) Maton, SSR, EST-SSR, ISSR

## Abstract

Cardamom (*Elettaria cardamomum* (L.) Maton) is a major export crop in Guatemala; however, its genetic basis remains largely unexplored. This study aimed to evaluate the genetic diversity and differentiation among 288 cardamom accessions from the Northern Transversal Strip, the country’s primary production area. Eleven molecular markers (SSR, ISSR, and EST-SSR) were used to generate multilocus profiles analyzed under a dominant model. Genetic diversity revealed average values of Shannon’s index (I = 0.316) and expected diversity (h = 0.207), with SSR markers providing the highest values (I = 0.364, h = 0.233). Bayesian and hierarchical analysis identified three genetic groups (K = 3). The relatively low diversity observed is consistent with the introduction history of this crop in Guatemala, human-driven selection, and historical bottlenecks caused by Cardamom Mosaic Virus and thrips infestations. Despite these constraints, private and high-frequency bands were detected across genetic groups, offering potential for marker-assisted selection. These findings provide the first genetic baseline for Guatemalan cardamom, supporting future breeding strategies aimed at improving resilience, productivity, and adaptation to climate change.

## 1. Introduction

Cardamom (*Elettaria cardamomum* (L.) Maton) is a perennial herbaceous plant belonging to the Zingiberaceae family. It is cultivated under shade and can reach heights of 2 to 5 m under ideal tropical, humid conditions [1,2]. The species develops from an underground rhizome system and can reproduce both sexually and vegetatively through rhizome division, giving rise to upright aerial shoots composed of overlapping leaf sheaths typically with elongated leaves [2]. Reproductive structures emerge from the rhizome, forming inflorescences that develop near or slightly above the soil surface [1,2]. As a cultivated crop, cardamom exhibits considerable morphological variation among accessions, particularly in vegetative growth and fruit characteristics. Importantly, cardamom is a tetraploid species, a condition that influences its genetic complexity and breeding strategies [2,3].

Originally a spice crop native to India, cardamom was introduced to Guatemala between 1910 and 1914, with the first seed were planted in Chinasayub, Cobán, Alta Verapaz [4]. Since then, its cultivation expanded to the southern coast and the Northern Transversal Strip, becoming a major contributor to Guatemala’s agricultural economy and providing livelihoods for more than 350,000 families [5,6]. Today, Guatemala is the world’s leading cardamom exporter, with approximately 169,429 hectares under cultivation and annual exports valued at USD 450 million [5,7].

Despite its economic importance, cardamom production in Guatemala has faced two major bottlenecks: outbreaks of Cardamom Mosaic Virus (CdMV) and infestations by the thrips, *Sciothrips cardamomi* [8,9]. These events, combined with the crop’s introduction from a limited genetic base, likely reduced genetic variability. Most production now occurs in the Northern Transversal Strip, in the areas of Alta Verapaz, Baja Verapaz, Quiché, Huehuetenango, and Izabal [7].

Understanding genetic structure in agricultural crops is essential for improving productivity, resilience, and adaptability [10]. Morphological studies have been conducted to assess diversity in cardamom; however, these traits are strongly influenced by environmental factors and human management, limiting their reliability [11]. Genetic diversity studies can identify accessions with resistance to pests and diseases, tolerance to climate change, and higher yield potential [12].

Research on cardamom genetics is scarce in Guatemala, despite its global leadership in production. Existing studies originate mainly from India and employ molecular markers such as Amplified Fragment Length Polymorphism (AFLP), Random Amplified Polymorphic DNA (RAPD), Simple Sequence Repeat (SSR), Inter-Simple Sequence Repeat (ISSR), and Expressed Sequence Tag–Simple Sequence Repeat (EST-SSR). For example, in 2015 Cyriac et al. evaluated the transferability of 79 SSR primers originally developed for *Amomum subulatum*, *Zingiber officinale*, and *Curcuma longa* to small cardamom (*Elettaria cardamomum*), finding that only 26 were transferable and revealed limited genetic variability [13].

Anjali et al. addressed the lack of a complete genetic map for cardamom by testing EST-SSR markers developed for *Curcuma longa*, reporting low to moderate diversity but confirming their usefulness for diversity and phylogenetic studies within Zingiberaceae [14]. In 2016, Anjali and colleagues introduced 26 ISSR markers to analyze intra-specific variation in 30 cardamom accessions, confirming the existence of genetic variability [15]. In 2017, Phadnis and Peter used RAPD markers to evaluate 11 genotypes, including Katte virus tolerant variety and wild types, successfully grouping them by geographic origin and plant type [16].

Cyriac et al. (2016) reported the first development of SSR markers specific to *Elettaria cardamomum* through selective hybridization enrichment, designing 58 primer pairs, of which 44 amplified expected products; however, only six provided polymorphic information [17]. Anisha et al. (2020) applied ISSR markers to 13 released varieties, including hybrids and selections, and differentiated nearly all evaluated genotypes [18]. More recently, Gaikwad et al. (2023) achieved the first de novo genome assembly for the variety Njallani Green Gold and developed 227,808 SSR primer pairs, validating 60 markers across 60 accessions. Their results revealed high allelic diversity and significant genetic admixture compared to previous studies [3].

Despite the crop’s economic relevance and its role in sustaining thousands of families, the genetic basis of Guatemalan cardamom remains largely unexplored. This knowledge gap limits future efforts to enhance productivity and resilience. Characterizing genetic diversity and population structure is essential for selecting accessions with tolerance to pests and diseases, adaptability to climate change, and improved yield potential. The present study addresses this need by evaluating the genetic diversity and genetic differentiation among 288 Guatemalan cardamom accessions using SSR, ISSR, and EST-SSR markers, integrating principal component analysis and using a Bayesian method.

## 2. Results

### 2.1. Genetic Diversity

In this study, a total of 11 molecular markers from three different types (SSR, ISSR, and EST-SSR) were employed to assess the genetic diversity of cultivated cardamom in the Northern Transversal Strip of Guatemala. Among these, SSR markers provided the highest values for genetic diversity (h) and Shannon’s diversity index (I), with values of 0.353 and 0.235, respectively (Table 1). Within the SSR markers, ECMG26 stood out by exhibiting the highest values for the number of alleles (Na = 7), Shannon’s index (I = 0.428), genetic diversity (h = 0.283), and polymorphism information content (PIC = 0.319), compared to the other SSR markers.

On the other hand, ISSR markers exhibited an average of 18 bands. The average values for Shannon’s index (I = 0.280) and genetic diversity (h = 0.180) were lower than those obtained with SSR markers (Table 1). Among the ISSR markers, S817 showed the highest total number of bands, while S820 demonstrated the greatest diversity, with values of I = 0.443 and h = 0.293 and S829 shows the highest polymorphism information content (PIC = 0.483) (Table 1).

Finally, EST-SSR markers showed average values that were similar to those of SSR markers, with Na = 6.5, I = 0.336, and h = 0.218. In this case, the marker CaSSR35 exhibited greater genetic diversity (I = 0.401, h = 0.273) and highest polymorphism information content (PIC = 0.295) than CaSSR26 (Table 1).

Among the bands recorded for each marker (Table S1), those associated with the highest diversity, based on the values of h and I, correspond to the markers previously identified as having the greatest genetic diversity.

### 2.2. Genetic Differentiation of Cardamom Accessions

**Figure 1:**
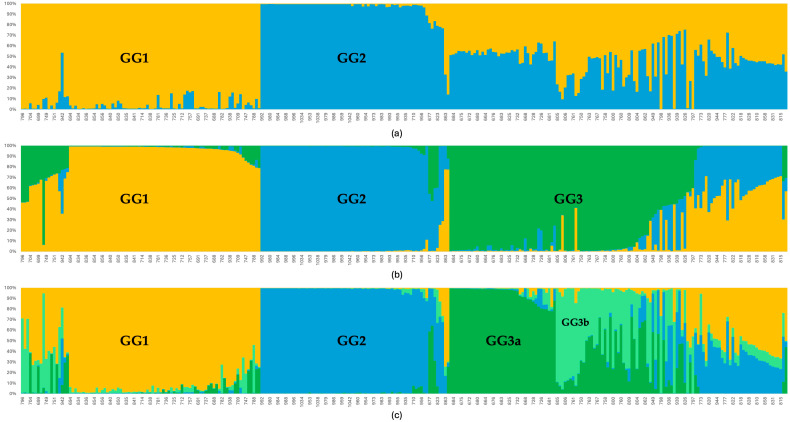
Bayesian method analysis of 288 Guatemalan cardamom accessions using microsatellite markers (SSRs, ISSRs, and EST-SSRs), performed with the software STRUCTURE version 2.3.4. The genetic groups were inferred assuming K = 2 (**a**), K = 3 (**b**), and finally K = 4 (**c**) to separate the GG3a and GG3b groups.

As a result of the genetic structure analysis conducted through a Bayesian method, three main genetic groups (K = 3) were identified among the 288 cardamom accessions from the Northern Transversal Strip of Guatemala (Figure 1). When assuming K = 2, two distinct genetic groups, designated as GG1 and GG2 (Genetic Group) were observed, while the remaining accessions were classified as admixed, indicating an unclear group assignment. However, under the assumption of K = 3, several of these previously admixed accessions were reassigned to a third distinct genetic group (GG3). A final attempt with K = 4 revealed further subdivision within GG3, resulting in the identification of two subgroups (GG3a and GG3b) (Table S2). The Mantel test revealed a weak but significant correlation between genetic and geographic distances (r = 0.081, *p* = 0.0001), indicating limited isolation by distance.

### 2.3. Hierarchical Clustering Analysis of Cardamom Accessions

**Figure 2:**
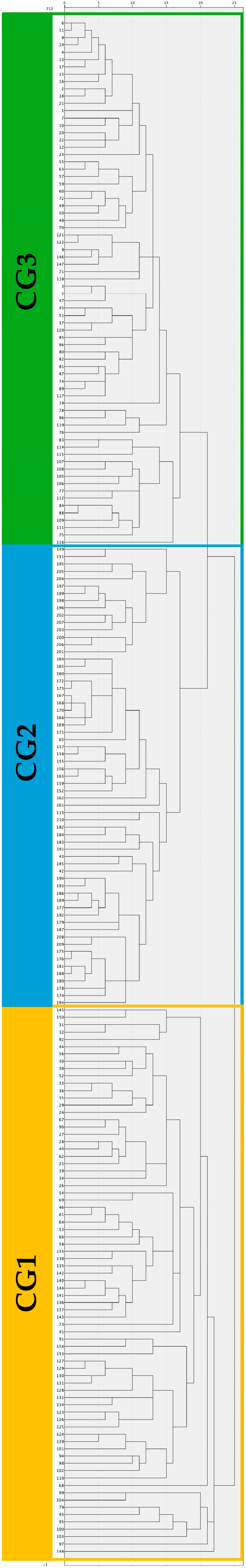
Hierarchical cluster analysis of Guatemalan cardamom accessions based on microsatellite markers (SSRs, ISSRs, and EST-SSRs) classified by Structure software for K = 3 (reconstructed genetic groups GG1-3).

A hierarchical clustering analysis was performed using average linkage between groups and Jaccard coefficient, excluding the unclassified (admixed) accessions (Figure 2). The dendrogram reveals three main clusters that include most of the accessions identified in each GG, as defined by the Structure program. The three clusters exhibited a relatively balanced distribution, with a similar number of accessions in each group. Specifically, Cluster 1 comprised 75 accessions (GG1), Cluster 2 included 63 accessions (GG2), and Cluster 3 contained 72 accessions (GG3).

### 2.4. Principal Component Analysis Using SSR, ISSR, and EST-SSR Markers to Study the Genetic Diversity of Cardamom

A Principal Component Analysis (PCA) was conducted using data from SSR, ISSR, and EST-SSR markers. Results showed that the three principal components accounted for 44.39% of the total variation (Table 2). Markers S817 (bands A to M) and S829 (bands A to O) were found to be relevant for the first two principal components (Table S3). Bands associated with marker S817 exhibited significant positive correlations exceeding 0.500 in both principal components, whereas bands from marker S829 showed a dual pattern: a significant negative correlation with the first principal component (values below −0.700) and a significant positive correlation with the second principal component, with values above 0.400 (Table S3). Marker S820, specifically band E, also showed positive significance in the second component. For the third principal component, marker S842 (bands A, B, C, E, J, K, and P) exhibited positive correlations with values above 0.200, along with representative bands such as ECM47a-C and S820 band E (Table S3).

**Figure 3:**
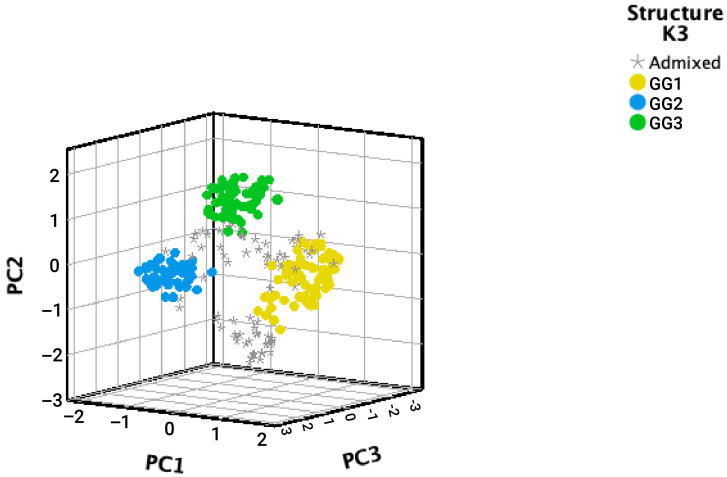
Association between inferred genetic groups structure (K = 3, reconstructed groups GG1-3) based on a Bayesian method and the first three principal components (PCs) derived from SSR, ISSR, and EST-SSR markers in 288 Guatemalan cardamom accessions.

Notably, accessions classified by Structure programs were differentiated by the first three PCs (Figure 3). GG1 is located in the positive PC1, meanwhile GG2 is in the negative PC1. GG3 has the most positive values for PC3. Accessions not clustered in a specific GG with a *qI* > 80% were positioned between the different GGs, representing a genetic mixture of the three groups.

### 2.5. Genetic Diversity Among Three Genetic Groups and Key Molecular Markers Associated with Their Differentiation

The main results of the AMOVA area summarize in Table 3 and also includes genetic diversity parameters for each group and relevant information on the molecular markers used to characterize the genetic groups.

The AMOVA, conducted among the identified reconstructed genetic groups (GG1, GG2, and GG3) and accessions classified as admixed (*qI* < 80%), indicated that 33% of the genetic variation occurred among groups (Table 3), while 67% is found within groups. The percentage of polymorphic loci was 76% for GG1, 50% for GG2, 84% for GG3, and 88% for admixed accessions, suggesting that GG2 exhibits lower genetic diversity compared to GG1, GG3, and even the admixed group.

Regarding the Shannon diversity index (I) and genetic diversity (h), higher values were observed in the accessions classified as admixed. The Nei genetic distance matrix indicated a greater level of differentiation between groups GG1 and GG2, with a value of 0.247, a distinction that is also evident in the first principal component (Figure 3). In contrast, a moderate degree of genetic relatedness is observed between GG1 and GG3 (0.139), and between GG2 and GG3 (0.148). This analysis confirms that the accessions classified as admixed exhibit common genetic parameters with the three GGs.

To characterize each of the GG, three types of bands were considered: private bands (PB), which are present exclusively in one group; missing bands (MB), which are absent in each group; and higher frequency bands (HF), which occur more frequently in one group compared to the others.

For example, in GG1, bands ECMG26-G, S817-U, and S842-R are private to this genetic group. In contrast, marker S829 lacks bands A to F and L to O in GG1 (Table S4).

In GG2, no private bands were identified; however, several missing bands were observed, including bands C, D, E, and F from marker CaSSR26; bands A to M and Q from marker S817; and bands C, E, F, L, M, and N from marker S836. Additionally, GG2 shows higher frequency bands in ECM47a-B, S829-N, and S829-O compared to the other groups (Table S4).

GG3 contains two private bands (S817-O and S817-T), one missing band (S842-Q), and one band with a higher frequency (S820-A) relative to the other groups. Although the admixed accessions represented a genetic mixture of the three GGs, they also exhibited unique characteristics, including two private bands (S842-N and S842-O) and one missing band (ECMG23-E) (Table S4).

## 3. Discussion

### 3.1. Global Genetic Diversity

Given the tetraploid nature of cardamom (*Elettaria cardamomum*), analyzing genetic diversity using SSR and EST-SSR markers presents significant technical challenges [3]. In genomes with four sets of chromosomes, it is not possible to accurately determine levels of heterozygosity or homozygosity at individual loci, unlike in diploid organisms where these markers are considered codominant [19]. Furthermore, allele overlapping and the potential presence of null alleles complicate the reliable identification of alleles at each locus [20]. Additionally, EST-SSR markers, which are derived from expressed regions of the genome, may be subject to selective pressures that influence their variability, adding another layer of complexity to the analysis [21]. For these reasons, this study adopted a conservative approach by analyzing the SSR and EST-SSR marker data under a dominant model, recording only the presence or absence of amplified alleles.

The same analytical model was applied to ISSR markers, which are inherently dominant and do not allow for the distinction between homozygous and heterozygous genotypes, regardless of ploidy level [22]. Collectively, the use of a dominant model across all marker types enabled a more robust and conservative interpretation of the genetic structure of cardamom groups.

Based on the three types of molecular markers analyzed (SSR, ISSR, and EST-SSR), a Shannon diversity index (I) of 0.316 and a genetic diversity (h) value of 0.207 were obtained. It is essential to account for the inherent characteristics of each marker, as they differ in the type of amplification they produce. The highest diversity value, both for the Shannon index (0.428) and genetic diversity (0.283), was observed with SSR markers. These results suggest that SSR markers are the most informative for evaluating the genetic diversity of the 288 cardamom accessions analyzed.

The average genetic diversity values obtained using SSR markers in this study (I = 0.364 and h = 0.233) were comparatively lower than those reported for other species within the same family Zingiberaceae. For instance, a study conducted on 57 accessions of *Etlingera elatior* populations in Malaysia reported diversity indices ranging from 0.92 to 0.98 using six SSR markers [23].

Similarly, the ISSR markers used in this study yielded lower diversity values (mean I = 0.280 and h = 0.180) indicating that ISSR markers reveal less information about the genetic diversity of the studied groups compared to SSR and EST-SSR markers. When compared to findings from a study conducted in Indonesia on accessions of *Zingiber officinale*, which employed 11 ISSR markers and reported diversity values ranging from 0.5 to 0.9 [24]. In the study conducted by Anajali et al., using the same markers, an average Shannon diversity index of 0.37 was obtained [15]. On the other hand, ISSR markers exhibited an average number of observed bands (18), which can be attributed to their ability to amplify regions between microsatellites and simultaneously target multiple loci [25].

In the case of EST-SSR markers, this study recorded average values of I = 0.331 and h = 0.214. These results are also lower than those reported in a study on turmeric species from the Republic of India, where 33 EST-SSR markers were developed and applied, yielding a Shannon diversity index of 0.49 and Nei’s gene diversity of 0.3314 [24]. In the study conducted by Anajali et al., a gene diversity value of 0.23 was reported [14].

It is important to consider that the comparative studies referenced in this analysis were conducted in regions and countries where the evaluated species are native. In contrast, the present study focuses on cardamom, a species introduced to Guatemala [24]. The relatively low genetic diversity observed may be influenced by this introduction process, which typically involves a genetic bottleneck due to limited founding material [26]. Furthermore, the genetic diversity detected in this study may also reflect the effects of human-mediated selection and cultivation practices that have shaped this crop over the past century [23,27].

Moreover, cardamom production has experienced two major genetic bottlenecks, one caused by a viral disease (Cardamom Mosaic Virus—CdMV) and another by the thrips *Sciothrips cardamomi* [8,9]. These events likely contributed to the low genetic diversity observed and may have played a role in shaping a crop population [28].

In studies of introduced species such as *Coffea arabica* L., similarly low genetic diversity values to those observed in the cardamom have been reported. For instance, in the study by Geleta et al., where SSR markers were used to assess the genetic diversity of *C. arabica* L. in Nicaragua, an average genetic diversity value of 0.353 was reported [29].

Among the markers analyzed, S829 exhibited the highest PIC value (0.483), exceeding those of the other marker types. Under the dominant marker model applied in this study, PIC represents the probability that two randomly selected individuals differ in band presence or absence at a given locus. The PIC value of 0.483 indicates high discriminatory power, considering that the theoretical maximum for dominant markers is 0.5. On average, ISSR markers were highly informative, with a mean PIC value of 0.289, whereas SSR and EST-SSR markers showed moderate informativeness, with mean PIC values of 0.240 and 0.238, respectively [30].

The presence of alleles with relatively high frequencies (>0.700) in SSR and EST-SSR markers across all evaluated accessions suggests a reduced allelic variation, which is a hallmark of a relatively low genetic diversity [31]. This pattern, particularly evident in SSR ECMG23, ECMG26, and ECM47a markers and EST-SSR CaSSR26, and CaSSR25 markers, may indicate that certain alleles have become fixed or nearly fixed within the accessions included in this study. Such fixation can result from several factors, including founder effects, genetic bottlenecks, and intense selection pressure, all of which are consistent with the introduction history and cultivation practices of cardamom in Guatemala.

Moreover, the dominance of specific alleles could reflect human-mediated selection, where traits linked to these alleles were favored during crop propagation [32]. Cardamom cultivation in Guatemala has been primarily disseminated across various regions through the exchange of rhizomes, where farmers have selected plants with the best phenological and productive characteristics, allowing this selection to prevail. The absence of this pattern in the bands of the ISSR markers, which typically target more variable intergenic regions, further supports the idea that functional or coding regions (targeted by SSR and EST-SSR markers) have undergone stronger selective constraints. This reinforces the hypothesis that the observed low genetic diversity is not only a consequence of historical bottlenecks but also of ongoing directional selection in cultivated cardamom genetic groups.

In the study conducted by Gaikwad et al., the researchers performed a *novo* assembly of the draft whole genome sequence of the cardamom variety Njallani Green Gold. Based on this genomic resource, they developed and evaluated novel SSR markers. Among the 60 SSR markers tested across 60 cardamom accessions, they reported an average of 14.57 alleles per locus, ranging from 4 to 30 alleles [3]. Other studies analyzing the genetic diversity of cardamom using SSR markers, EST-SSR markers, and ISSR markers have reported lower allele counts or lower total number of bands [14,15,17]. Specifically, Cyriac et al. found between 2 and 7 alleles per SSR locus, while Anajali et al. reported 1 to 7 polymorphic ISSR bands per primer [15,17].

Unlike Gaikwad et al., the present study did not use newly designed SSR markers. Instead, we employed previously published SSR, ISSR, and EST-SSR markers from earlier studies [14,15,17,21]. Even so, we observed a relatively high number of alleles and total number of bands: SSR markers revealed between 5 and 12 alleles, ISSR markers showed 11 to 24 bands, and EST-SSR markers exhibited up to 7 alleles. These results suggest that, despite using established markers, the genetic diversity detected in our accessions may be higher than previously reported. Nonetheless, although our findings indicate comparatively high allelic variation in the case of SSR and EST-SSR markers, the overall genetic diversity detected is still lower than that documented by the previously cited studies, also with ISSR markers.

### 3.2. Cardamom Genetic Differentiation Among Accessions Based on Molecular Markers

This study represents the first analysis of the genetic differentiation among accessions outside the species’ native range. Specifically, it is the first to characterize how cardamom genetics have been structured following more than a century of introduction and distribution in Guatemala with a selected number of accessions. Due to the absence of historical records documenting the introduction process, the specific varieties initially brought into the country remain unknown. However, it is established that cardamom was first introduced in the department of Cobán, Alta Verapaz, and subsequently spread across the southern coastal region and into the Northern Transversal Strip of Guatemala [4,7]. This expansion likely involved multiple genetic bottlenecks, as previously discussed.

The genetic group structure analysis conducted in this study allowed for the classification of 288 cardamom accessions from the Northern Transversal Strip into three distinct genetic groups, based on data obtained using SSR, ISSR, and EST-SSR markers. It is plausible that these three genetic clusters are associated with the cardamom varieties originally introduced into Guatemala, or that, throughout more than a century of cultivation and under diverse selective pressures, genetic differentiation has occurred within the country. However, further studies integrating molecular and morphological parameters are required to confirm these associations and to determine whether such differentiation corresponds to distinct varietal groups. Notably, no clear geographic correlation was found among the three genetic groups (Table S5), indicating that group differentiation is not driven by spatial distribution. This interpretation is supported by the weak but significant Mantel correlation (Mantel r = 0.081, *p* = 0.0001), which shows that geographic distance contributes only marginally to the genetic structure of the accessions.

To further explore the genetic relationships among these accessions, a hierarchical clustering analysis was performed using average linkage and Jaccard distance, excluding admixed individuals that could not be clearly assigned to any of the three genetic groups. This analysis was also used to verify the selection of the number of groups inferred by the Bayesian method in STRUCTURE, ensuring consistency between both methods [33]. The resulting dendrogram (Figure 2) reveals a relatively balanced distribution of accessions across the three genetic groups, suggesting a uniform distribution of genetic diversity. The balanced distribution may indicate a shared genetic origin among accessions, similar selection pressures across the regions where cardamom is cultivated (especially due to the events caused by the CdMV on the South Coast), or a combination of both.

The AMOVA results reveal a significant genetic structure among groups, with 33% of the molecular variance attributed to differences among groups. While the majority of variance (67%) still occurs within groups. Our findings are consistent with those reported by Kumar & Kumar (2018) for *Lepidium sativum* L., where the exact same percentages of within- and among-group variation were observed. Based on these values, the authors indicated that the species possesses a significant genetic structure [34]. The presence of admixed individuals (those not clearly grouped within the three main groups) suggests that this differentiation is not absolute and points to ongoing genetic mixing or shared ancestry. These accessions also exhibited higher values for the Shannon diversity index (I) and genetic diversity (h), likely due to their shared genetic characteristics with all three established genetic groups, which contribute to increased diversity within this group. Although controlled crosses between cardamom varieties have not been documented in the field, vegetative propagation and informal selection practices by farmers may have facilitated genetic recombination among distinct genotypes.

In contrast to the present study, the research conducted by Gaikwad et al., which employed SSR markers to evaluate 60 cardamom accessions from a varietal collection in the Republic of India, enabled the classification of these accessions into five major genetic groups [3]. Furthermore, their analysis revealed genetic affinities with the ‘Malabar’, ‘Mysore’, and ‘Vazhukka’ varietal types, highlighting the importance of identifying these affinities in future studies of cardamom accessions in Guatemala [3].

### 3.3. Key Molecular Markers Associated with Cardamom Genetic Groups in Guatemala

To identify the most representative bands from the SSR, ISSR, and EST-SSR marker data for the 288 cardamom accessions, a principal component analysis (PCA) was conducted. The ISSR markers S817 and S829 exhibited the greatest influence on the first two principal components, while ISSR marker S820 showed a moderate contribution to the second component. In the third principal component, ISSR marker S842 was also significant. Notably, this component highlighted the relevance of ECM47a, a marker distinct from the ISSR group, underscoring its potential importance in explaining genetic variation. Collectively, these markers capture key patterns of genetic diversity and help define the genetic structure of cardamom in Guatemala.

Given that this study represents one of the first efforts to assess the genetic diversity of cardamom in Guatemala and has identified three genetically distinct groups, it is essential to further explore and characterize the specific markers or alleles found in this study that are representative of each genetic group. Such markers could serve as valuable tools in future studies on the genetic diversity of Guatemalan cardamom.

The analysis of bands presented in Table S4, including private, absent, and high-frequency bands, enabled the identification of distinct genetic characteristics among the three cardamom genetic groups (GG1, GG2, and GG3) from the Northern Transversal Strip of Guatemala. The presence of private bands in GG1 and GG3, along with the absence of specific bands in GG2, provides clear evidence of genetic differentiation among these groups. Accessions classified as “admixed” exhibited a combination of genetic features from all three groups, along with unique bands, suggesting a history of genetic admixture and potential gene flow among groups. These findings are critical for informing the development of conservation strategies, sustainable management practices, and genetic improvement programs for cardamom cultivation in Guatemala.

## 4. Materials and Methods

### 4.1. Plant Material

**Figure 4:**
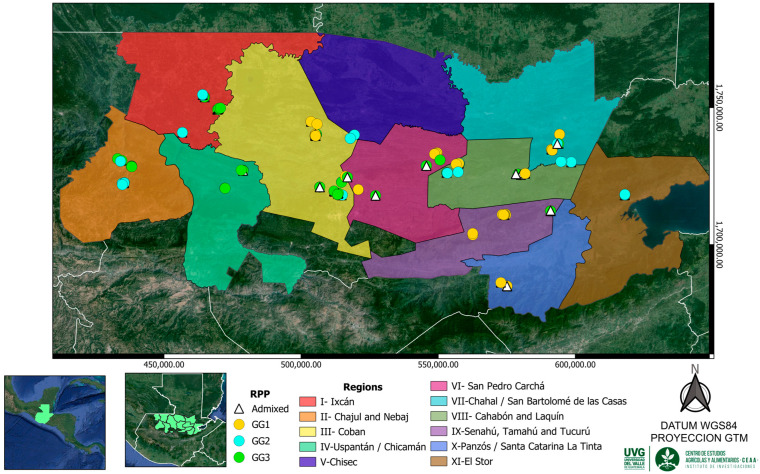
Collection sites of a sample of 288 accessions in the cardamom production regions at the Northern Transversal Strip of Guatemala classified by the reconstructed groups (K = 3) obtained by the Structure program.

This research study was conducted in the departments of Alta Verapaz, Quiché, and Izabal, located within the Northern Transversal Strip of Guatemala, an area currently recognized as the country’s primary cardamom-producing region [7]. The three departments were organized into eleven productive regions, each comprising one or more municipalities (Figure 4). Within these regions, a total of 288 cardamom accessions were sampled and distributed as follows: 29 accessions from Region I (Ixcán), 25 from Region II (Chajul and Nebaj), 60 from Region III (Cobán), 12 from Region IV (Chicamán and Uspantán), 18 from Region V (Chisec), 21 from Region VI (San Pedro Carchá), 27 from Region VII (Chahal and Fray Bartolomé de Las Casas), 48 from Region VIII (Cahabón and Lanquín), 21 from Region IX (Senahú, Tamahú, and Tucurú), 18 from Region X (Panzós and Santa Catarina La Tinta), and finally, 9 accessions from Region XI (El Estor) (Table S6).

At each collection site (Figure 4), cardamom accessions were selected based on the farmer’s criteria regarding which plant was considered the most productive within their plot. For this study, an accession was defined as a single cardamom plant sampled from a cultivated site, selected by the producer as representative or agronomically superior, and assigned a unique identification code. For each selected plant, a sample was collected consisting of a segment of the rhizome. This sample was transported to the Universidad del Valle de Guatemala (Central Campus), located in the department of Guatemala, where a living cardamom collection was established using the collected accessions. Additionally, another sample from each selected plant was collected. These samples consisted of young leaf tissue used for DNA extraction, resulting in a total of 288 samples.

### 4.2. Molecular Markers Analysis

#### 4.2.1. Molecular Markers Selection

A total of 11 molecular markers belonging to three categories were used in this study (Table 4): Simple Sequence Repeat (SSR) markers described by Cyriac et al. (2016), Inter-Simple Sequence Repeat (ISSR) markers developed by Anjali et al. (2016), and Expressed Sequence Tag–Simple Sequence Repeat (EST-SSR) markers reported by Anjali et al. (2015) [14,15,17]. A pilot test was conducted to verify the amplification efficiency of these markers, and eleven were selected based on the presence of clear and well-defined bands observed on a polyacrylamide gel [24].

#### 4.2.2. DNA Extraction

DNA extraction was carried out using young leaf tissue from each cardamom accession. A total of 0.1 g of leaf material was collected and stored at −20 °C for at least 20 min. The tissue was then macerated in a solution containing 200 µL of 5% 2-mercaptoethanol, 150 µL of 5% sodium metabisulfite, 100 µL of 10% PVP and 50 µL of sterile distilled water. Following maceration, DNA was extracted using the cetyltrimethylammonium bromide (CTAB) protocol described by Doyle and Doyle [35]. Upon completion of the extraction, 1 µL of RNase was added to each sample. DNA integrity was assessed by electrophoresis on a 1% agarose gel, and DNA concentration and purity were determined using a spectrophotometer Thermo GENESYS™ 10 UV-Vis (Thermo Electron Scientific Instruments Corporation, Madison, WI, USA).

#### 4.2.3. DNA Amplification

For SSR-type markers, a 25 µL PCR reaction was performed using a final concentration of 1X PCR Buffer, 2 mM MgCl_2_, 0.1 mM dNTPs, 0.2 µM of each primer, 0.5 U of GoTaq^®^ DNA Polymerase (Promega^®^, Madison, WI, USA) and 1 µL of DNA. DNA amplification was carried out with an initial denaturation step at 94 °C for 5 min, followed by 40 cycles of denaturation at 94 °C for 30 s, primer annealing at 60 °C for 45 s, and extension at 72 °C for 1 min. A final extension step was performed at 72 °C for 10 min.

For ISSR marker analysis, the PCR reaction was carried out in a 26 µL volume containing a final concentration of 1X PCR Buffer with MgCl_2_, 0.2 mM dNTPs, 0.2 µM of each primer, and 1 U of GoTaq^®^ DNA Polymerase (Promega^®^) and 1 µL of DNA. Amplification was initiated with one cycle at 94 °C for 2 min, followed by 35 cycles of denaturation at 94 °C for 30 s, primer annealing for 1 min at the specific melting temperature of each primer, and extension at 72 °C for 2 min. A final extension step was performed at 72 °C for 7 min.

For EST-SSR marker analysis, the PCR reaction was performed in a 25 µL volume containing a final concentration of 1X PCR Buffer with MgCl_2_, 0.2 mM dNTPs, 0.2 µM of each primer, and 1 U of GoTaq^®^ DNA Polymerase (Promega^®^) and 1 µL of DNA. DNA amplification was carried out under the same thermal cycling conditions described for ISSR markers. For all three marker types (SSR, ISSR, and EST-SSR), DNA amplification was performed using an Eppendorf^®^ Mastercycler^®^ Nexus Thermal Cycler (Eppendorf SE, Hamburg, Germany).

#### 4.2.4. Band Visualization

PCR products were separated by vertical electrophoresis [36,37,38]. For this, a 10% polyacrylamide gels (10 × 10 cm, 0.75 mm thickness) were used, following the methodology proposed by Creste et al. (2001) and the protocol development for BIO-RAD^®^ with modifications [39,40,41]. The gels were prepared by mixing 6.6 mL of distilled water, 2.4 mL of 5X sodium borate (SB) buffer and 3 mL of bis-acrylamide (29:1) solution. Polymerization was initiated by adding 20 µL of TEMED and 20 µL of 10% ammonium persulfate (APS), followed by a 30 min incubation at room temperature. Once polymerization was complete, the gels were placed in the electrophoresis tank and submerged in 1X SB buffer. Prior to loading, each sample was prepared by mixing 4 µL of PCR product with 1 µL of stopmix (containing formamide, EDTA, xylene-cyanol, and bromophenol blue). The samples were then loaded onto the gel, alongside 2 µL of 100 bp DNA ladder (Promega^®^) for PCR products obtained with SSR and EST-SSR markers, while a 1 kb DNA ladder (Promega^®^) was used for ISSR marker products. Electrophoresis was conducted at a constant voltage of 160 V for 60 min.

Following electrophoresis, bands were visualized using silver staining according to the protocol described by Creste et al. (2001), with modifications [39]. Gels were initially immersed in 150 mL of fixing solution (10% *v*/*v* glacial acetic acid) for 20 min; this solution was then collected and reserved to terminate the reaction later. Subsequently, gels were rinsed three times for two minutes each with deionized water. Staining was performed by incubating gels for 20 min in 150 mL of staining solution containing 0.1% (*w*/*v*) silver nitrate and 1.5 mL·L^−1^ of 37% formaldehyde. After staining, gels were briefly rinsed with deionized water (10 s) to remove excess silver nitrate. Band development was carried out by immersing gels in 150 mL of developer solution composed of 3% (*w*/*v*) sodium carbonate, 1.5 mL·L^−1^ of 37% formaldehyde, and 0.0005% (*w*/*v*) sodium thiosulfate, pre-cooled to 4 °C. Development continued until bands reached the desired intensity, after which the reaction was stopped by reapplying the 10% acetic acid fixing solution [40].

For each accession and each marker analyzed, the observed band sizes (Figures S1–S3) were estimated using Gel Analyzer 23.1.1 software (Version 20), and the initial data were recorded in a Microsoft Excel spreadsheet (Version 16.98) [42]. Once the complete dataset for all accessions and markers was compiled, banding patterns were characterized through manual visual inspection, based on the number of bands, their spatial distribution, and molecular size. Additionally, only high-quality, intensely stained bands were considered for scoring, while ambiguous signals were excluded to maintain data reliability. Each distinct band was assigned a letter along with the corresponding marker name. Considering the tetraploid nature of cardamom, all markers (SSR, EST-SSR, and ISSR) were analyzed under a dominant model. Subsequently, a binary data matrix was constructed based on the amplification patterns, where ‘1’ indicated the presence and ‘0’ the absence of a given band (Table S7) [38,43,44].

### 4.3. Data Analysis

#### 4.3.1. Group Genetic and Structure Analysis

To investigate the genetic differentiation among the accessions, STRUCTURE version 2.3.4 was used [45,46]. This software applies a Bayesian method under an admixture model with correlated allele frequencies to assign individuals to genetic groups based on multilocus genotype data [46]. Analyses were conducted using K values ranging from 1 to 15, with 10 replicate runs per K value. Each run consisted of a burn-in length of 10,000 iterations followed by 100,000 Markow chain Monte Carlo (MCMC) interactions. The CLUMPAK platform (Kopelman et al., 2015) was used to align and summarize replicate runs, ensuring consistency in cluster assignment across runs [47]. The resulting Q matrix provided membership coefficients (Q values), representing the proportion of each individual’s genome assigned to each inferred cluster. Individuals were assigned to a specific cluster when their Q value exceeded 0.80; otherwise, they were considered admixed [48].

#### 4.3.2. Genetic Diversity Analysis

Genetic diversity parameters were estimated using GenAlEx version 6.5, based on a binary matrix constructed under a dominant scoring model [49]. Considering the tetraploid nature of cardamom, all marker types (SSR, EST-SSR, and ISSR) were scored as presence/absence data, where each amplified allele (for SSR and EST-SSR markers) or band (for ISSR markers) was coded as 1 or 0 [3]. From this matrix, the following parameters were calculated: frequencies of alleles for SSR/EST-SSR markers and bands for ISSR markers, where p represents band frequency and q = 1 − p, the number of different alleles (Na) for SSR and EST-SSR markers, total number of bands for ISSR markers, the Shannon diversity index, and expected genetic diversity (h) [49,50]. The polymorphic information content (PIC) was calculated under a dominant marker model. PIC values were estimated for each locus using the formula PIC = 2f(1 − f), where f represents the frequency of band presence across accessions [30,51]. Additionally, group structure inferred from STRUCTURE analysis was incorporated to compare genetic groups (GGs). The number of private bands per genetic group was identified, and an Analysis of Molecular Variance (AMOVA) was conducted to evaluate genetic differentiation among and within groups [52,53].

The relationship between genetic and geographic distances was assessed using a Mantel test [54]. Individual-based genetic distances were calculated using Jaccard’s binary distance, and geographic distances were calculated in kilometers from latitude and longitude coordinates using the Haversine formula [55,56]. Significance was evaluated using Pearson correlation coefficients with 9999 permutations [57]. Analyses were performed in R (version 4.4.1) using the vegan, geosphere, readxl, and dplyr packages [58,59,60,61,62].

IBM SPSS Statistics version 29.0.02 was employed to perform a Principal Component Analysis (PCA) based on a covariance matrix, to identify the main components contributing to genetic variation [63,64].

A hierarchical cluster analysis was also conducted using the between-groups linkage method (average linkage), with Jaccard’s binary similarity coefficient as the dissimilarity measure [65]. This method is particularly appropriate for presence/absence data, such as binary matrices derived from microsatellite markers.

SPSS was further used to generate graphical representations of the PCA and clustering results. A principal component analysis (PCA) was conducted using data from SSR, ISSR, and EST-SSR markers to reduce dimensionality and identify the bands most relevant to the genetic diversity of the 288 cardamom accessions evaluated.

## 5. Conclusions

This study provides the first published evidence on the genetic diversity and genetic differentiation among cardamom cultivated in the Northern Transversal Strip of Guatemala, offering a baseline resource for future breeding and conservation efforts. By analyzing 288 cardamom accessions with 11 molecular markers (SSR, ISSR, and EST-SSR), we detected overall low levels of genetic diversity, consistent with the species’ introduction into Guatemala, human-driven selection, prevailing agricultural practices, and two major genetic bottlenecks caused by susceptibility to Cardamom Mosaic Virus (CdMV) and thrips infestations. Among marker types, SSRs were the most informative, while ISSRs and EST-SSRs provided complementary insights. Despite the reduced genetic variation, the analysis revealed three genetic groups that could potentially be categorized as varieties. However, it is necessary to perform an association analysis between these reconstructed genetic groups and the morphological characteristics of each group to formally establish them. Furthermore, a relatively balanced distribution of accessions among these groups was evident. Finally, this study recommends the use of selected ISSR and SSR markers for the identification and classification of cardamom accessions within these three genetic groups.

## Figures and Tables

**Table 1:** Genetic diversity parameters per marker—including allele frequencies, number of different alleles (Na), Shannon diversity index (I), and expected genetic diversity (h)—in 288 cardamom accessions from Guatemala.

Microsatellite Type	Primer	Na	Total Number of Bands	I	h	uh	PIC *
SSR	ECMG23	6	-	0.236	0.186	0.146	0.147
	ECMG26	7	-	0.428	0.283	0.288	0.319
	ECMG28	5	-	0.350	0.216	0.219	0.224
	ECM47a	12	-	0.397	0.255	0.259	0.272
EST-SSR	CaSSR26	7	-	0.272	0.163	0.165	0.180
	CaSSR35	6	-	0.401	0.273	0.277	0.295
ISSR	S817	-	24	0.189	0.121	0.122	0.280
	S829	-	15	0.239	0.155	0.157	0.483
	S842	-	21	0.347	0.223	0.227	0.230
	S820	-	11	0.443	0.293	0.298	0.327
	S836	-	21	0.180	0.107	0.109	0.123

* PIC values were calculated assuming a dominant marker model based on band presence (1) and absence (0).

**Table 2:** Principal Component Analysis followed by marker correlation to assess genetic diversity and differentiation among 288 Guatemalan cardamom accessions using SSR, ISSR, and EST-SSR markers.

Molecular Marker	Eigenvalue		
	PC1	PC2	PC3
Value	3.577	2.822	1.511
Contribution rate (%)	20.076	15.836	8.479
Accumulated contribution rate (%)	20.076	35.912	44.392

**Table 3:** AMOVA results, genetic diversity parameters, and marker-based characterization of three cardamom reconstructed genetic groups (GG1, GG2, GG3) based on SSR, ISSR, and EST-SSR markers in 288 Guatemalan accessions.

Information		Cardamom Genetic Group			
		GG1	GG2	GG3	Admixed
Percentage of Molecular Variance Among Groups		33%			
Percentage of Molecular Variance Within Groups		67%			
Percentage of Polymorphic Loci		76%	50%	84%	88%
I ^1^		0.274	0.202	0.298	0.391
h ^2^		0.174	0.131	0.185	0.257
uh ^3^		0.176	0.133	0.187	0.260
Pairwise Group Matrix of Nei Genetic Distance	GG1	0.000	-	-	-
	GG2	0.247	0.000	-	-
	GG3	0.139	0.148	0.000	-
	Admixed	0.043	0.105	0.110	0.000

^1^ I = Shannon’s information index; ^2^ h = Nei’s gene diversity; ^3^ uh = unbiased gene diversity.

**Table 4:** Molecular markers (SSR, ISSR, and EST-SSR) used to assess the genetic diversity of 288 Guatemalan cardamom accessions.

Microsatellite Type	Microsatellite Name	Primer Sequence (5′–3′)		Repeat Motif	Annealing Temperature (°C)	Expected Product Size (bp)
		Forward	Reverse			
SSR ^1^	ECMG23	TCTAAAGGAGGGAACATGGATA	GGTTAAGATGAAGGCAAAAGAG	(TTTG)4	58.4	236
	ECMG26	CAATTACTCAGCGAAACCTGTG	GAGCTTCTAAACTGGTGCGAAT	(CA)11	60.3	206
	ECMG28	TGTTCAGAGGAGTCAGCAGGTA	GCCTCAAACTTCTTGTCCATCT	(TATC)5TA	61.2	148
				(TTTG)4		
				TC(TATT)3		
	ECM47a	CTCCCTCTTCCTCTTTTCTTTT	CCATATCACAGACATAGCAAGG	(CT)17TCAA(TC)	59.3	137
EST-SSR ^2^	CaSSR26	CTGGGATGGGTATCTACAATGA	CAGTGAGTCCACAGAAAGCAAT	(ATC)6	60.3	299
	CaSSR35	CTTGACAGGACAGCAACAGAAC	CGATGACAGAAGAGAGAGAGCA	(AGC)6	62.1	156
ISSR ^3^	S817	CACACACACACACACAT		NA	50	200–2000
	S829	ACACACACACACACC		NA	46.1	200–2000
	S842	GAGAGAGAGAGAGAGAYC		NA	53.9	200–2000
	S820	CACACACACACACACAA		NA	50	200–2000
	S836	AGAGAGAGAGAGAGAGYC		NA	53.9	200–2000

^1^ SSR markers described by Cyriac et al. (2016); ^2^ EST-SSR markers reported by Anjali et al. (2015); and ^3^ ISSR markers developed by Anjali et al. (2016) [14,15,17].

## Data Availability

The data are contained within the article or Supplementary Materials.

## Supplementary Materials

The following supporting information can be downloaded at: https://www.mdpi.com/article/10.3390/plants15040655/s1, Table S1: Microsatellites genetic diversity per band; Table S2: STRUCTURE K2 K3 K4; Table S3: PC Eigen vectors and correlation; Table S4: Band frequency of cardamom genetic groups; Table S5: Dendrograms without admixed; Table S6: Collection database; Table S7: Allele database; Figures S1–S3: Examples of PAGE gels by marker.
