# Supplementary material for: Genetic Diversity and Differentiation Among Guatemalan Cardamom (Elettaria cardamomum (L.) Maton) Accessions
Source: Plants (Basel). 2026 Feb 20;15(4):655. doi: 10.3390/plants15040655 (PMC12944064; doi:10.3390/plants15040655)
Supplement: Supplementary file 1 [file plants-15-00655-s001.zip › Figure S1 - 3 Examples of PAGE gels by marker.pdf]

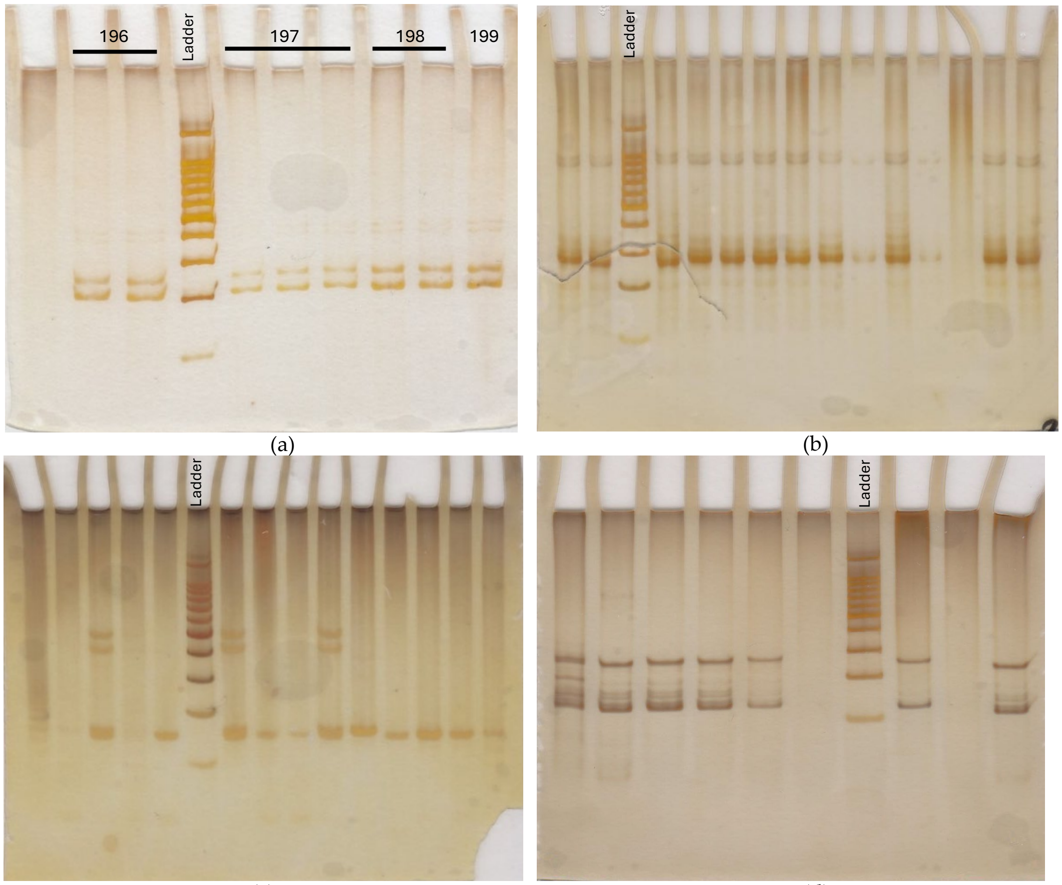


(a) (b) (c) (d)

Figure S1. Example of acrylamide electrophoresis at 10% of molecular marker type SSR with a 100 bp DNA ladder. (a) corresponds at primer ECMG26 with some samples duplicated and triplicated; (b) Primer ECMG23; (c) ECMG28 and (d) ECM47a with samples not replicated.

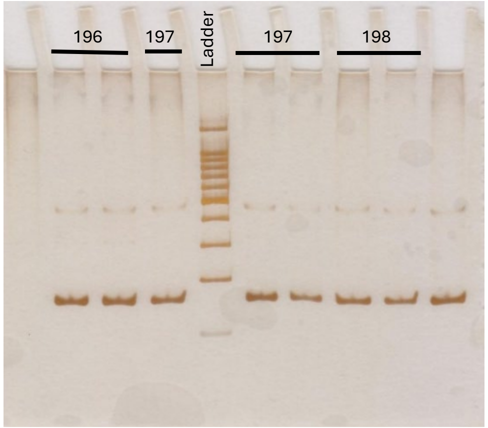


(a)

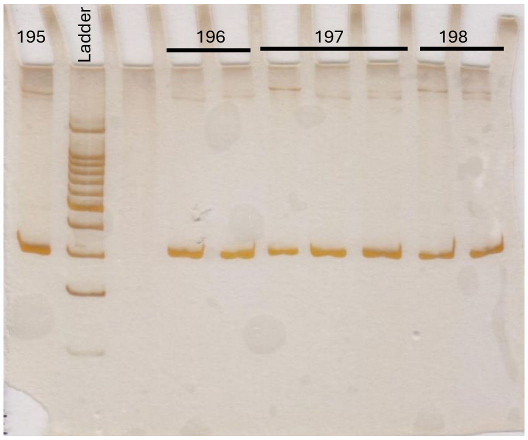


(b)

Figure S2. Examen of acrylamide electrophoresis at 10% of molecular marker type EST-SSR with a 100 bp DNA ladder. (a) corresponds at primer CaSSR35 and (b) CaSSR26, with some samples duplicated and triplicated.

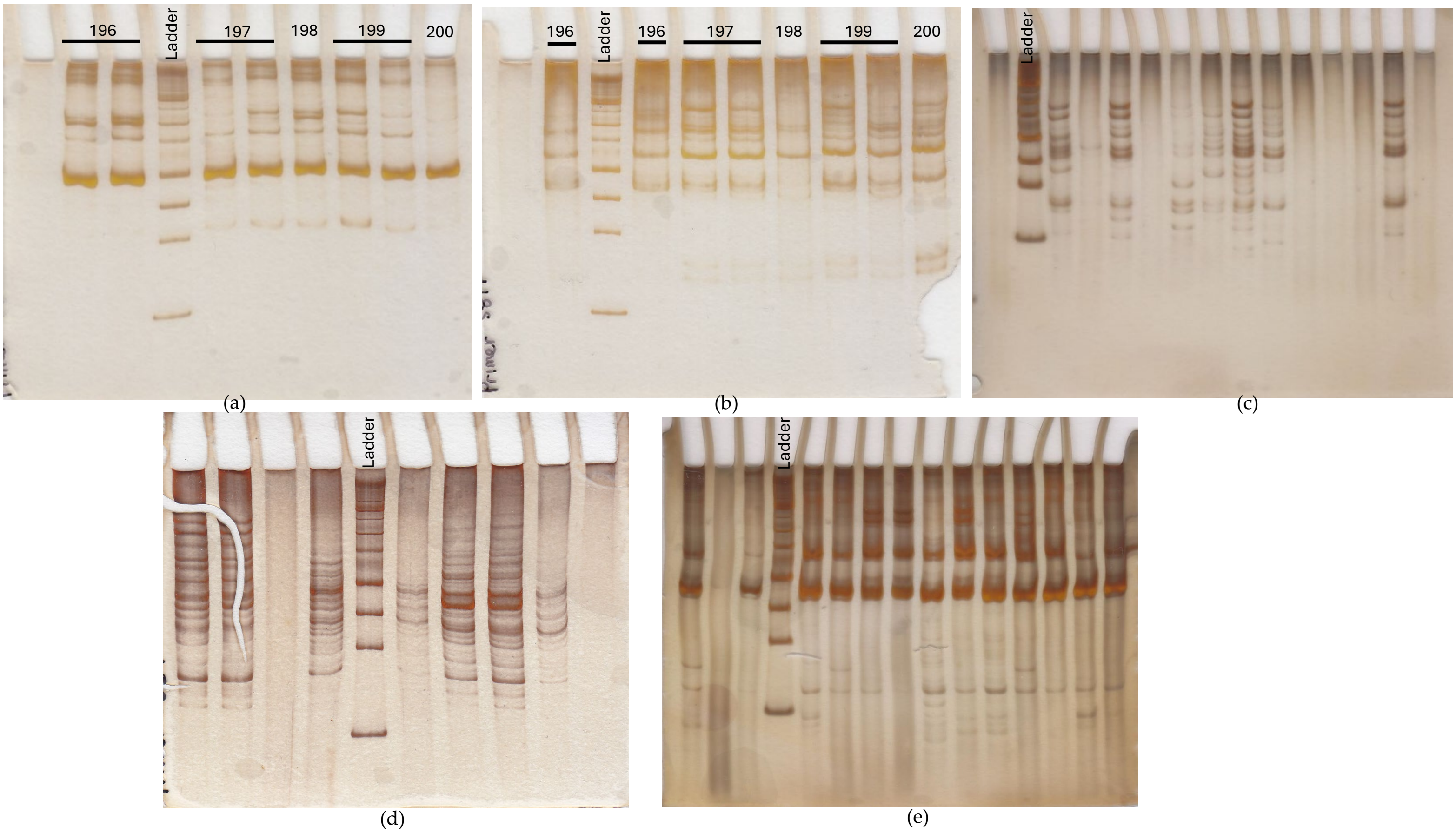


Figure S3. Examen of acrylamide electrophoresis at 10% of molecular marker type ISSR with a 1 kb DNA ladder. (a) corresponds at primer S820; (b) S817, with some samples in duplicated; (c) Primer S836, (d) S829 and (e) S842 with samples not replicated.
